# GLP-1 Receptor Agonists and Coronary Arteries: From Mechanisms to Events

**DOI:** 10.3389/fphar.2022.856111

**Published:** 2022-03-08

**Authors:** Aurélie Pahud de Mortanges, Eldem Sinaci, Dante Salvador, Lia Bally, Taulant Muka, Matthias Wilhelm, Arjola Bano

**Affiliations:** ^1^ Faculty of Medicine, University of Bern, Bern, Switzerland; ^2^ Institute of Social and Preventive Medicine, University of Bern, Bern, Switzerland; ^3^ Department of Cardiology, Inselspital, Bern University Hospital, University of Bern, Bern, Switzerland; ^4^ Department of Diabetes, Endocrinology, Nutritional Medicine, and Metabolism, Inselspital, Bern University Hospital, University of Bern, Bern, Switzerland

**Keywords:** GLP-1 receptor agonists, diabetes, coronary artery disease, acute and chronic coronary syndromes, atherosclerosis, coronary microcirculation

## Abstract

**Objective:** Glucagon-like peptide 1 receptor agonists (GLP-1 RAs) lower plasma glucose through effects on insulin and glucagon secretion and by decelerating gastric emptying. GLP-1 RAs have many beneficial effects beyond glycemic control, including a protective role on the cardiovascular system. However, underlying mechanisms linking GLP-1 RAs with coronary artery disease are complex and not fully elucidated. In this mini-review, we discuss these mechanisms and subsequent clinical events.

**Data Sources:** We searched PubMed and Google Scholar for evidence on GLP-1 RAs and coronary events. We did not apply restrictions on article type. We reviewed publications for clinical relevance.

**Synopsis of Content:** In the first part, we review the current evidence concerning the role of GLP-1 RAs on potential mechanisms underlying the development of coronary events. Specifically, we discuss the role of GLP-1 RAs on atherosclerosis and vasospasms of epicardial coronary arteries, as well as structural/functional changes of coronary microvasculature. In the second part, we summarize the clinical evidence on the impact of GLP-1 RAs in the prevention of acute and chronic coronary syndromes and coronary revascularization. We conclude by discussing existing gaps in the literature and proposing directions for future research.

## Introduction

Acute coronary syndromes (ACS), such as unstable angina pectoris (uAP), non-ST-elevated myocardial infarction (NSTEMI), and ST-elevated myocardial infarction (STEMI), and chronic coronary syndromes (CCS) replacing the term “stable angina” in 2019 ESC guidelines, are prevalent conditions with major contributions to morbidity and mortality (Neumann et al., 2020; Collet et al., 2021; Kurdi, 2021). Patients with type 2 diabetes mellitus (T2DM) have higher incidences of coronary events compared with individuals with normoglycemic levels (Leon and Maddox, 2015). Glucagon-like peptide 1 receptor agonists (GLP-1 RAs), including structural analogues of human endogenous GLP-1 (albiglutide, dulaglutide, liraglutide, and semaglutide) and exendin-based agents (efpeglenatide, exenatide, and lixisenatide), are antidiabetic medications that share common glucose-lowering mechanisms, such as augmentation of glucose-induced insulin secretion, suppression of glucagon, and deceleration of gastric emptying (Heuvelman et al., 2020; Pradhan et al., 2020). Beyond glycemic control, GLP-1 RAs have a beneficial role on the cardiovascular system through antagonizing the development of cardiovascular risk factors, such as obesity, and exerting protective effects on the heart and macro- and microcirculation (Clarke et al., 2018; Garg et al., 2019; Wang et al., 2020; Giugliano et al., 2021a).

**FIGURE 1:**
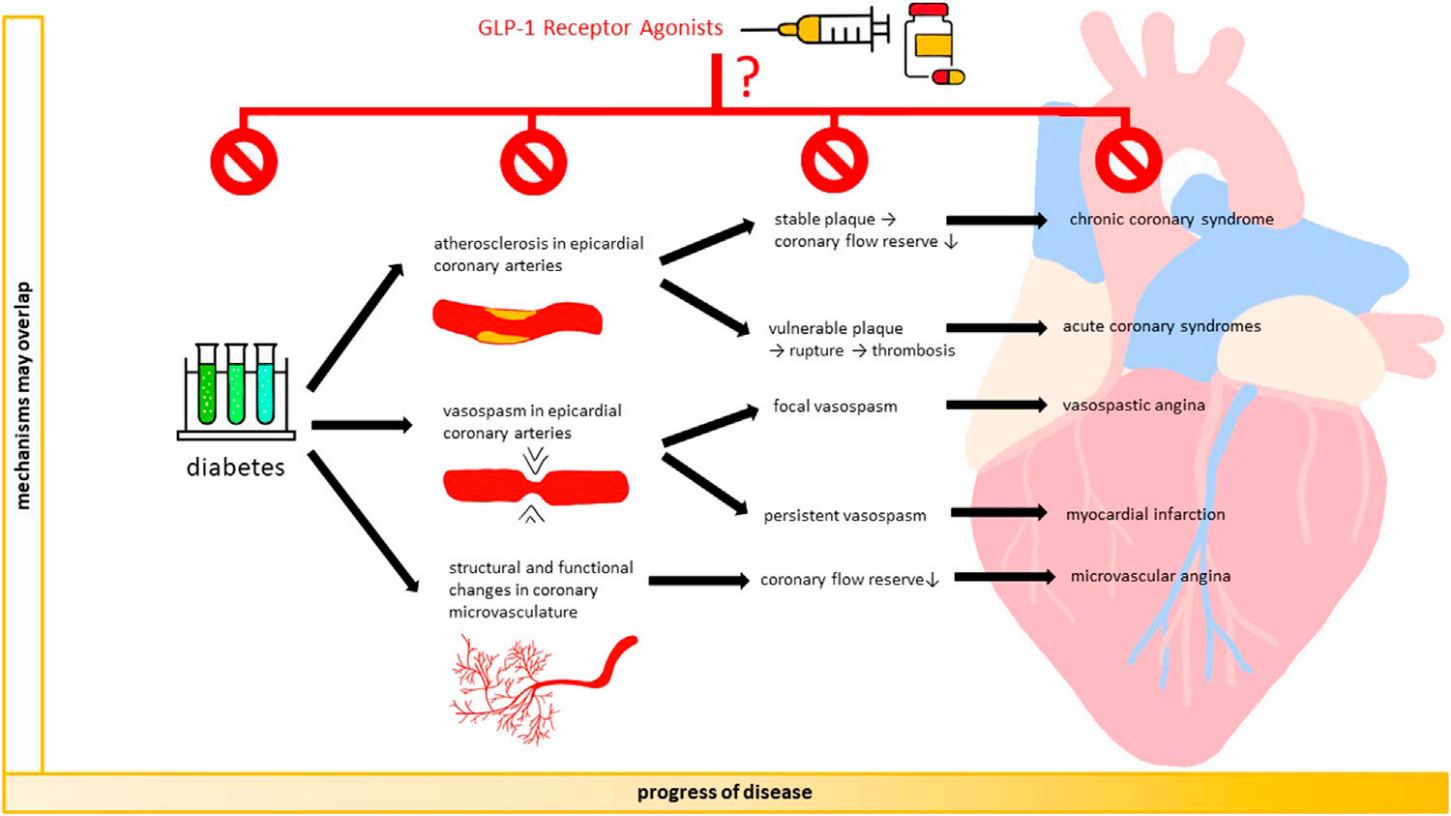
GLP-1 RAs and coronary arteries: from mechanisms to events. GLP-1 receptor agonists may prevent the development and progression of coronary atherosclerosis, vasospasm of epicardial coronary arteries, and structural/functional changes in coronary microvasculature. By occurring alone or concomitantly, these mechanisms reduce coronary blood flow and may result in coronary events, including acute coronary syndrome, chronic coronary syndrome, vasospastic angina, and microvascular angina.

Although recent reviews have described the role of GLP-1 RAs on cardiovascular risk in general, there is a lack of evidence synthesis on local actions of GLP-1 RAs in coronary arteries and the link of these mechanisms to coronary events (Garg et al., 2019; Heuvelman et al., 2020). In this narrative mini-review, we discuss the association of GLP-1 RAs with atherosclerosis and vasospasm of epicardial coronary arteries, as well as structural/functional changes of coronary microcirculation. We focused on these processes because they are crucial to the development of coronary events, including ACS and CCS and vasospastic angina and microvascular angina (MVA) (Figure 1). Furthermore, we provide an overview on GLP-1 RAs and the risk of coronary events.

## Methods

We searched PubMed and Google Scholar for mechanistic and clinical evidence about GLP-1 RAs and coronary events. We used the following keywords: GLP-1, GLP-1 receptor agonist, exenatide, liraglutide, lixisenatide, semaglutide, albiglutide, dulaglutide, efpeglenatide, coronary artery disease, coronary atherosclerosis, endothelial dysfunction, inflammation, body weight, blood pressure, lipids, aggregation, coagulation, coronary vasospasm, coronary vasodilation, coronary microvascular, acute coronary syndrome, myocardial infarction, unstable angina, stable angina, chronic coronary syndrome, vasospastic angina, microvascular angina, coronary revascularization. In this mini-review, we prioritized the reporting of the most relevant articles regarding the role of GLP-1 RAs on coronary atherosclerosis, vasospasm, microcirculation, and subsequent coronary events. We excluded articles about vessels other than coronary arteries.

## Glucagon-Like Peptide 1 Receptor Agonists and Pathways Underlying Coronary Events

### Glucagon-Like Peptide 1 Receptor Agonists and Atherosclerosis of Epicardial Coronary Arteries

Atherosclerosis is a chronic condition that can progressively occlude epicardial coronary arteries. Stable atherosclerotic plaques induce inadequate blood supply to the myocardium, resulting in CCS. Unstable plaques can lead to rupture, thrombosis, and subsequent ACS (Figure 1).

Although investigations in animals are scarce, studies among humans have suggested that GLP-1 RAs directly mitigate the development of coronary atherosclerosis through several mechanisms (Garg et al., 2019) (Table 1). First, GLP-1 RAs can improve endothelial function and reduce oxidative stress in coronary arteries (Erdogdu et al., 2010, 2013; Kapadia et al., 2021). Accordingly, GLP-1 receptors were detected in human coronary artery endothelial cells (hCAECs) (Nyström et al., 2004). Exendin-4, GLP-1 (7-36), and GLP-1 (9-36) increased DNA synthesis in hCAECs with subsequent cell proliferation;(Erdogdu et al., 2010) while exendin-4 and GLP-1 (7-36) protected hCAECs against lipoapoptosis (Erdogdu et al., 2013). These mechanisms were mediated by the GLP-1 receptor and involved protein kinase A (PKA), phosphatidylinositol-3-kinase/protein kinase B (PI3K/Akt), and endothelial nitric oxide synthase (eNOS) pathways. In another study performed in hCAECs, exendin-4, liraglutide, albiglutide, and lixisenatide led to a decrease in endoplasmic reticulum stress by downregulating the unfolded protein response (Kapadia et al., 2021).

**TABLE 1:** Effect of GLP-1 RAs on mechanisms leading to coronary events.

Study	Compound^a^	Type of study	Results
**Coronary atherosclerosis**			
Endothelial Dysfunction and Oxidative Stress			
Nyström et al. (2004)	GLP-1 (7-36) amide	study in humans; 12 men with T2DM, obesity, and coronary artery disease; 10 healthy men	GLP-1 receptors were detected in coronary artery endothelial cells
Erdogdu et al. (2010)	Exendin-4	*in vitro* study; hCAECs	PKA-PK13/Akt-eNOS ↑
	GLP-1 (7–36)		DNA synthesis proliferation of hCAECs ↑
	GLP-1 (9–36)		
Erdogdu et al. (2013)	Exendin-4	*in vitro* study; hCAECs	lipoapoptosis ↓
	GLP-1 (7-36)		
Kapadia et al. (2021)	Exendin-4	*in vitro* study; hCAECs	ER stress ↓
	Liraglutide		
	Albiglutide		
	Lixisenatide		
Inflammation and Leukocyte Adhesion			
Garczorz et al. (2018)	Exenatide	*in vitro* study; hCAECs	activation of NF-κB ↓
			activation of adhesion molecules ICAM and VCAM ↓
Noyan-Ashraf et al. (2013)	Liraglutide	*in vitro* study; hCASMC	lipotoxicity ↓
Plaque Burden and Size			
Piotrowski et al. (2013)	endogenous GLP-1 concentrations	study in humans; 303 participants undergoing CT angiography for chest pain	GLP-1 concentrations significantly associated with total coronary plaque burden
Hamal et al. (2020)	Semaglutide	study in humans (double-blind placebo controlled RCT); 140 participants with T2DM and coronary atherosclerosis	study in progress
Birudaraju et al. (2021)	Semaglutide	study in humans (double-blind placebo controlled RCT); 140 participants with T2DM	study in progress
**Coronary Vasospasms**			
Xiong et al. (2019)	GLP-1^b^	animal study; healthy rats	dilatation of pre-constricted coronary arteries
		*in vitro* study, rCASMS	enhancement of the K_ATP_ channel dependence of effect on endothelium
Sukumaran et al. (2020)	Liraglutide	animal study; rats, lean and obese on high salt diet	coronary vessel internal diameter ↑
**Impairment of Coronary Microvascular Function**			
Sukumaran et al. (2020)	Liraglutide	animal study; rats, lean and obese on a high salt diet	improvement of small vessel dilatory response to acetylcholine
Wang et al. (2013)	Exenatide	animal study; rats with diabetes	preservation of microvascular integrity attenuation of lanthanum nitrate across endothelial cells → protection of microvascular barrier function
Wang et al. (2020)	GLP-1^b^ ± Insulin	study in humans; 15 participants without diabetes; with obesity	MBV and MBF ↑
			MFV ↓ if no insulin added
Subaran et al. (2014)	GLP-1^b^	study in humans; 26 participants without diabetes	MBV and MBF ↑
			MFV ↓
Clarke et al. (2018)	GLP-1 (7-36)	study in humans; 21 participants with mixed diabetes status and stable angina	basal microcirculatory resistance ↓
Aetesam-ur-Rahman et al. (2021)	GLP-1 (7-36) amide ± Theophylline	study in humans; 41 participants with mixed diabetes status and stable angina	basal microvascular resistance ↓
Faber et al. (2015)	Liraglutide	study in humans; 24 participants with diabetes	no difference in CFR assessed by Doppler echocardiography between treatment and control arms
Nilsson et al. (2019)	GLP-1 (7-36)	study in humans; 12 participants without diabetes, with obesity	no difference in CFVR assessed by Doppler echocardiography
Suhrs et al. (2019)	Liraglutide	study in humans; 33 women without diabetes; with overweight and coronary microvascular dysfunction	no difference in CFVR assessed by Doppler echocardiography before and after treatment

a The study by Piotrowski et al. measured endogenous GLP-1 concentrations. All other studies used exogenous compounds.

b GLP-1 fragment not specified.

Note: The studies presented in this table describe direct effects of GLP-1 RAs, on macro- and microvascular coronary arteries. Studies concerning indirect mechanisms linking GLP-1 RAs, to coronary events are not included.

Abbreviations: Akt protein kinase B, CFR, coronary flow reserve; CFVR, coronary flow velocity reserve; CT, computed tomography; DNA, deoxyribonucleic acid; eNOS, endothelial nitric oxide synthase; ER, endoplasmic reticulum, GLP-1, glucagon-like peptide 1, GLP-1 RAs, glucagon-like peptide 1 receptor agonists; hCAECs, human coronary artery endothelial cells; hCASMC, human coronary artery smooth muscle cells; ICAM, intracellular adhesion molecules; K_ATP_, channel ATP-sensitive potassium channel, MBF, microvascular blood flow; MBV, microvascular blood volume; MFV, microvascular flow velocity; NF-κB, nuclear factor kappa-light-chain-enhancer of activated B cells, PI3K phosphoinositide 3-kinase, PKA, protein kinase A; ROS, reactive oxygen species; rCASMC, rat coronary artery smooth muscle cells, T2DM, type 2 diabetes mellitus; VCAM, vascular cell adhesion molecule.

Second, GLP-1 RAs can reduce inflammation in coronary arteries. An *in vitro* study in hCAECs found that exenatide decreased inflammation by reducing activation of nuclear factor kappa-light-chain-enhancer of activated B cells (NF-κB), adhesion molecules intracellular adhesion molecules (ICAM) and vascular cell adhesion molecules (VCAM), but not P-selectin (Garczorz et al., 2018). The modulation of NF-κB by GLP-1 RAs can be mediated by counteracting the changes in methylation levels of NF-κB promotor region that were induced by high glucose levels (Scisciola et al., 2020). One can thus assume that GLP-1 RAs can affect the epigenetic mechanisms regulating the expression of genes involved in vascular changes. Another experimental study showed that liraglutide decreases lipotoxicity of palmitic acid in human coronary smooth muscle cells (CSMCs) (Noyan-Ashraf et al., 2013).

Third, GLP-1 RAs potentially affect coronary plaque characteristics. An observational study performed among 303 participants with and without T2DM who underwent computed tomography (CT) angiography due to chest pain, reported a positive association of endogenous GLP-1 levels with coronary plaque burden (Piotrowski et al., 2013). It could thus be assumed that in a state of high cardiovascular risk, the body tries to limit disease progression by increasing the secretion of the protective agent GLP-1. To our knowledge, there are two double-blind placebo controlled randomized controlled trials (RCTs) on the effect of semaglutide on coronary plaque burden, size and composition among patients with diabetes in progress (Hamal et al., 2020; Birudaraju et al., 2021). However, more high-quality studies evaluating the role of other GLP-1 RA compounds are needed. Furthermore, it can be hypothesized that GLP-1 RAs reduce atherosclerotic plaque vulnerability in diabetes by promoting sirtuin 6 (SIRT6) expression, increases in collagen content, adiponectin secretion, and its anti-inflammatory effects through adaptor protein PH domain and leucine zipper containing 1 (APPL1) signaling pathways (Balestrieri et al., 2015; Barbieri et al., 2017). Future studies need to elucidate the exact role of GLP-1 RAs on coronary plaque stability and progression.

In addition to direct actions in coronary arteries, GLP-1 RAs can prevent the formation and progression of coronary atherosclerosis through indirect mechanisms, such as maintaining glycemic control, losing weight, decreasing systolic blood pressure, obtaining beneficial effects from lipid levels, inhibiting platelet activation, reducing systemic inflammation and levels of circulating BNP (Sun et al., 2015a, 2015b; Song et al., 2015; Cameron-Vendrig et al., 2016; Barale et al., 2017; Mazidi et al., 2017; Sardu et al., 2018; Sternkopf et al., 2020; Trujillo et al., 2021). However, in-depth discussion of these factors is beyond the scope of our review.

### Glucagon-Like Peptide 1 Receptor Agonists and Vasospasm of Epicardial Coronary Arteries

Spasms in the smooth muscle layer of the coronary artery wall can be focal or diffuse, resulting in vasospastic angina or myocardial infarction (MI), respectively. Few studies in animals have indicated that GLP-1 RAs may affect the tone of coronary arteries through inhibition of vasospasm or promotion of vasodilatation (Xiong et al., 2019; Sukumaran et al., 2020). In *ex vivo* rat models, GLP-1 reversed the contraction of excised coronary artery rings induced by a thromboxane receptor agonist (Xiong et al., 2019). In another study conducted in a rat model of metabolic syndrome, chronic treatment with liraglutide showed a greater increase in coronary vessel internal diameter than vehicle control (Sukumaran et al., 2020).

The vasodilatory effects of GLP-1 RAs can be mediated through GLP-1 receptors or alternative pathways. GLP-1 receptors were detected through immunohistochemistry in CSMCs of mice, suggesting that GLP-1 RAs may act directly on CSMCs (Ban et al., 2008; Richards et al., 2014). Other putative mechanisms of action of GLP-1 RAs on vascular smooth muscles may include intracellular alterations in activities of protein kinase C, Rho-Kinase, myosin light chain kinase, as well as calcium handling, G-proteins, and ion channels (Lanza et al., 2011). In particular, it was suggested that GLP-1 may increase potassium-ATP currents, thereby increasing membrane potential of smooth muscle cells and preventing depolarization and contraction (Xiong et al., 2019). On the other hand, the vasodilatory effects of GLP-1 RAs can also be mediated through amelioration of endothelial function, which in turn affects vascular smooth muscle tone through release of nitric oxide, endothelium-derived hyperpolarizing factor, and prostacyclins (Sandoo et al., 2010). The vasodilatory effects were not observed after denuding the endothelium from the rat coronary arteries, thus suggesting endothelium-dependent effects (Xiong et al., 2019).

Among humans, GLP-1 receptors were detected through western blotting in CSMCs and coronary artery endothelial cells (Nyström et al., 2004; Goto et al., 2011). However, there is scarce evidence reported for the exact underlying pathways linking GLP-1 and GLP-1 RAs to the focal and diffuse spasms of coronary arteries. Moreover, it remains unclear whether GLP-1 RA-related changes in coronary diameter differ between atherosclerotic and non-atherosclerotic coronary arteries and if effects are dose-dependent.

### Glucagon-Like Peptide 1 Receptor Agonists and Structural/Functional Changes in Coronary Microvasculature

Structural and functional abnormalities in coronary microcirculation decrease myocardial blood flow, further resulting in myocardial ischemia and MVA (Crea et al., 2014; Taqueti and Di Carli, 2018). Changes in microcirculation can occur alone or coexist with coronary atherosclerosis or vasospasm (Crea et al., 2014).

Studies in animals suggest that GLP-1 RAs modulate coronary microvascular function (CMF). A study evaluating the effects of liraglutide treatment in lean and obese rats on a high salt diet found improved dilatory response of microvessels to acetylcholine (Sukumaran et al., 2020). Another study performed in rats with diabetes found the antidiabetic drug exenatide preserved cardiac microvascular integrity and attenuated diffusion of lanthanum nitrate across endothelial cells, indicating protective properties on cardiac microvascular barrier function (Wang et al., 2013).

Among humans, some studies suggest an association between GLP-1 RAs and CMF, while others report no association. In a recent study, fifteen adult volunteers with obesity received infusions with either GLP-1, GLP-1+insulin, or saline placebo (Wang et al., 2020). Measurements of cardiac microvascular blood volume (MBV), microvascular flow velocity (MFV), and microvascular blood flow (MBF) were performed by contrast enhanced echocardiography. Among participants receiving only GLP-1 infusion, MBV and MBF increased significantly, while MFV decreased. The addition of insulin, which usually acts as a vasodilatory agent, did not increase MBV or MBF compared with GLP-1 alone, which suggests the presence of microvascular insulin resistance and the insulin-independent effects of GLP-1 on cardiac microvasculature. Similar results were obtained in another study conducted among 26 healthy young volunteers (Subaran et al., 2014). After 150 min of GLP-1 infusion, contrast enhanced echocardiography revealed an increase in cardiac MBV by 57%, in MBF by 47%, and a concomitant decrease in MFV. The change in MBV can be considered substantial, given that even small increases in tissue MBV markedly improve oxygen supply when tissue total blood flow is limited, such as in the presence of coronary atherosclerosis. Overall, the findings indicated that GLP-1 enhance cardiac microvascular perfusion. Furthermore, a study among 21 patients awaiting percutaneous coronary intervention (PCI) for stable angina found that basal microcirculatory resistance (MCR) decreased after GLP-1 administration (Clarke et al., 2018). These results were supported by another study performed among 41 patients with stable angina undergoing PCI (Aetesam-ur-Rahman et al., 2021). In this study, the effect of GLP-1 on basal MCR persisted after administration of theophylline—an adenosine receptor antagonist—indicating that the vasodilatory effects of GLP-1 in human coronary arteries are unlikely to be mediated by adenosine. Other studies did not find beneficial effects of GLP-1 or GLP-1 RAs for cardiac microvascular health when it was measured by transthoracic echocardiography to evaluate coronary flow reserve (CFR), which is also sometimes referred to as coronary flow velocity reserve (CFVR) (Samady and Hung, 2013; Faber et al., 2015; Nilsson et al., 2019; Suhrs et al., 2019). In a single-blinded cross-over RCT that included twenty-four patients with T2DM without previous coronary artery disease (CAD), 10 weeks of liraglutide treatment had no effect on CFR when compared to no treatment (Faber et al., 2015). In an open-label, proof-of-concept study that included thirty-three women with overweight, coronary microvascular dysfunction, and without diabetes, CFVR did not change after a 12 week period of liraglutide treatment when compared to the control period (Suhrs et al., 2019). In another study of twelve participants with overweight, GLP-1 (7-36) had no effects on CFVR when compared to saline control (Nilsson et al., 2019).

Heterogeneity of study results among humans could have various explanations. First, measurements of CMF vary across studies. CFR, which represents the maximal dilator response to an arteriolar vasodilator, is frequently applied but carries the disadvantage of representing the effects of macro- and microvascular disease (Taqueti and Di Carli, 2018; Masi et al., 2021). Abnormalities in CFR can only be attributed to coronary microvasculature if no flow-limiting CAD in epicardial arteries is detected upon coronary angiography (Taqueti and Di Carli, 2018). Coronary angiography—a difficult procedure to perform systematically in study settings—was carried out in only one of the aforementioned studies evaluating CF(V)R (Suhrs et al., 2019). To rule out coronary macrovascular disease, other studies used treadmill exercise testing or information from medical histories, which might be less reliable than coronary angiography (Faber et al., 2015; Nilsson et al., 2019). Second, numbers of study participants were small (n ≤ 41). Third, participants’ clinical characteristics varied across studies. So far, it is unknown whether the effects of GLP-1 RAs on coronary microvasculature differ by age, sex, comorbidities, and participant cardiovascular risk profiles. Fourth, time and method of GLP-1 RA administration varied largely across studies, and it still remains unclear whether there are differences between the efficacy of short-term GLP-1 infusion and longer-term oral administration of GLP-1 RAs (Subaran et al., 2014; Faber et al., 2015; Clarke et al., 2018; Nilsson et al., 2019; Suhrs et al., 2019; Wang et al., 2020; Aetesam-ur-Rahman et al., 2021).

Further studies are needed with adequate numbers of participants using highly accurate measures of CMF, such as positron emission tomography or cardiac magnetic resonance, to verify the hypothesis of GLP-1 and GLP-1 RAs affecting CMF. Future research also needs to explore potential differences according to patient clinical characteristics, type of GLP-1 RA compound, as well as time, method, and dosage of GLP-1 RA administration.

## Glucagon-Like Peptide 1 Receptor Agonists and Coronary Events

### Composite Outcomes Including Coronary Events

Coronary events form a part of cardiovascular composite outcomes, such as cardiovascular mortality and major adverse cardiovascular events (MACE). The latter is commonly defined as a composite of cardiovascular mortality, non-fatal MI, and non-fatal stroke (i.e., 3-point MACE), which sometimes also involves uAP (i.e., 4-point MACE). GLP-1 RAs have positive effects on the prevention of cardiovascular mortality. Two recent meta-analyses of eight cardiovascular outcome trials (CVOTs) and several other meta-analyses that included non-CVOT studies estimated the reduction in cardiovascular mortality varied between 12 and 15% (Zheng et al., 2018; Zhu et al., 2020; Giugliano et al., 2021b; Palmer et al., 2021; Sattar et al., 2021) (Table 2). Accordingly, meta-analyses found that the risk of MACE was lower among the GLP-1 RA group than among the control group, estimating a risk reduction between 12 and 14% (Zhu et al., 2020; Giugliano et al., 2021b; Sattar et al., 2021). In a recent meta-analysis of eight CVOTs that included 60,080 participants, the overall risk of MACE was reduced by 14% (Hazard Ratio [HR] = 0.86, 95% Confidence Interval [CI] = 0.79–0.94) (Giugliano et al., 2021b). The beneficial effect of GLP-1 RAs tended to be greater among participants with previous cardiovascular disease (CVD) (HR = 0.84, 95%CI = 0.79–0.90) than among those without CVD (HR = 0.94, 95%CI = 0.83–1.06), though there was no statistically significant difference between groups. One possible explanation for the putative effect modification from CVD can be that the reduction in MACE needs more time to become apparent among patients without previous CVD (Zelniker et al., 2019). Furthermore, this effect might not be captured by clinical studies with limited follow-up periods.

**TABLE 2:** Overview of studies reporting on GLP-1 RAs and the risk of coronary events.

Study	Compound	Type of study	Number of study participants	Comparator	Diabetes status	Previous CV disease status^d^	Outcome and quality of evidence
CV mortality							
Sattar et al. (2021)	various GLP-1 RAs	MA of 8 CVOTs	60,080	placebo	T2DM	mixed	**HR: 0.87 (0.80–0.94)** ^a^
Giugliano et al. (2021b)	various GLP-1 RAs	MA of 8 CVOTs	60,080	placebo	T2DM	mixed	**HR: 0.87 (0.78–0.96)** ^b^
Palmer et al. (2021)	various GLP-1 RAs	network MA of RCTs	not specified	placebo	T2DM	very low to very high risk of cardiovascular outcomes	**OR: 0.88 (0.80–0.96)** _a_
Zhu et al. (2020)	various GLP-1 RAs	umbrella review and MA of systematic reviews and MAs of RCTs	60,556	placebo	T2DM, prediabetes or at high risk of diabetes	mixed	**RR: 0.87 (0.81–0.94)** _a_
Zheng et al. (2018)	various GLP-1 RAs	network MA of RCTs	40,022	placebo or no treatment	T2DM	mixed	**HR: 0.85 (0.77–0.94)**
MACE							
Sattar et al. (2021)	various GLP-1 RAs	MA of 8 CVOTs	60,080	placebo	T2DM	mixed	**HR: 0.86 (0.80–0.93)** ^c^
Giugliano et al. (2021b)	various GLP-1 RAs	MA of 8 CVOTs	60,080	placebo	T2DM	mixed	**HR: 0.86 (0.79–0.94)** ^b^
Zhu et al. (2020)	various GLP-1 RAs	umbrella review and MA of systematic reviews and MAs of RCTs	59,999	placebo	T2DM, prediabetes or at high risk of diabetes	mixed	**RR: 0.88 (0.83–0.92)** ^a^
Myocardial Infarction							
Sattar et al. (2021)	various GLP-1 RAs	MA of 8 CVOTs	60,080	placebo	T2DM	mixed	**fatal and non-fatal MI: HR: 0.90 (0.83–0.98)** ^a^
Zhu et al. (2020)	various GLP-1 RAs	umbrella review and MA of systematic reviews and MAs of RCTs	165,858	placebo	T2DM, prediabetes or at high risk of diabetes	mixed	**fatal and non-fatal MI: RR: 0.92 (0.86–0.99)** ^a^
Palmer et al. (2021)	various GLP-1 RAs	network MA of RCTs	not specified	placebo	T2DM	very low to very high risk of cardiovascular outcomes	**non-fatal MI: OR: 0.92 (0.85–0.99)** ^a^
Giugliano et al. (2021b)	various GLP-1 RAs	MA of 8 CVOTs	60,080	placebo	T2DM	mixed	non-fatal MI: HR: 0.91 (0.81–1.01)^b^
Huang et al. (2017)	Exenatide and liraglutide	MA of RCTs	361	placebo	mixed	acute myocardial infarction	reduction of infarct size
Unstable Angina Pectoris							
Zhu et al. (2020)	various GLP-1 RAs	umbrella review and MA of systematic reviews and MAs of RCTs	56,004	placebo	T2DM, prediabetes or at high risk of diabetes	mixed	RR: 1.06 (0.93–1.21)^a^
Zheng et al. (2018)	various GLP-1 RAs	network MA of RCTs	25,966	placebo or no treatment	T2DM	mixed	HR: 0.94 (0.76–1.16)
Chronic Coronary Syndrome							
Myat et al. (2021)	Liraglutide	RCT	22	placebo	1 patient with T2DM; 21 without	>70% stenosis in main epicardial coronary; normal resting state ECG; ST-segment depression upon exercise tolerance test	no change in magnitude of ST-segment depression at peak exercise during sequential exercise tolerance test
Revascularization							
Gerstein et al. (2021)	Efpeglenatide	CVOT (AMPLITUDE-O)	4,076	placebo	T2DM	mixed	HR: 0.93 (0.69–1.26)
Marso et al. (2016)	Liraglutide	CVOT (LEADER)	9,340	placebo	T2DM	mixed	HR: 0.91 (0.80–1.04)
Gerstein et al. (2019)	Dulaglutide	CVOT (REWIND)	9,901	placebo	T2DM	mixed	incidence of 6% in treatment group and 6.3% in placebo group, *p* = 0.46
Microvascular Angina							
Suhrs et al. (2019)	Liraglutide	open-label, proof of concept study	33	control period without intervention	no diabetes	coronary microvascular dysfunction	improvement of 1/5 dimensions (physical limitation score) of microvascular angina symptoms

Bold values indicate statistically significant results.

a Quality of evidence: Studies report their evidence to be of high certainty.

b This meta-analysis states all included studies to be of high quality.

c The quality of evidence for this outcome was not classified as high due to heterogeneity deriving from the ELIXA, trial. However, when ELIXA, is excluded from the analysis, this outcome can also be classified to have high quality of evidence.

d Previous cardiovascular disease: The term “mixed” refers to a population that comprises individuals with and without previous ASCVD.

Abbreviations: AP, angina pectoris; CCS, chronic coronary syndrome; CV, cardiovascular, GLP-1 RA, Glucagon-like peptide 1 receptor agonist; HR, hazard ratio; MACE, major adverse cardiovascular event, MA meta-analysis; MI, myocardial infarction; OR, odds ratio; RR, risk ratio; TIMI, thrombolysis in myocardial infarction, T2DM, type 2 diabetes mellitus.

Selection of reported studies: Studies reported in the text and above in the table were selected according to data relevancy and currency. If recent meta-analyses were available, these were reported preferentially. If not, CVOTs, RCTs, or other types of studies were considered.

### Acute Coronary Syndromes

*Myocardial infarction:* Emerging evidence supports beneficial effects of GLP-1 RAs on MI risk. A CVOT meta-analysis and an umbrella review of meta-analyses of RCTs reported that GLP-1 RAs reduce the risk of combined fatal and non-fatal MI when compared to placebo (HR = 0.90, 95%CI = 0.83–0.98; Risk Ratio [RR] = 0.92, 95%CI = 0.86–0.99, respectively) (Zhu et al., 2020; Sattar et al., 2021). The meta-analysis included eight CVOTs comprising 60,080 patients with T2DM (Sattar et al., 2021). The umbrella review included thirty-one RCTs involving 165,858 participants with either T2DM, prediabetes, or at high risk of diabetes (Zhu et al., 2020). Certainty of evidence was high. Another network meta-analysis of RCTs, including participants with T2DM, found that GLP-1 RAs decreased the risk of non-fatal MI (Odds Ratio [OR] = 0.92, 95%CI = 0.85–0.99) when compared to placebo or standard care (Palmer et al., 2021). Of note, it has been suggested that the risk reduction related to GLP-1 RAs can be smaller for MI than for stroke (Sattar et al., 2021). These risk differences might be explained by additional mechanisms beyond the actions of GLP-1 RAs on vasculature, including the chronotropic effects of GLP-1 RAs which may blunt cardiac protection (Garg et al., 2019). GLP-1 RAs may also be used in the management of MI. A meta-analysis of four RCTs found that GLP-1 RAs reduced infarct size when compared to placebo (weighted mean difference in final infarct size in % of the left ventricle = −4.06, 95%CI = −7.64—-0.47) (Huang et al., 2017).

*Unstable angina pectoris:* An umbrella review of meta-analyses of RCTs including 56,004 patients with diabetes, prediabetes, and at high risk of diabetes, found no association between GLP-1 RAs and the risk of uAP (RR = 1.06, 95%CI = 0.93–1.21) when compared to placebo (Zhu et al., 2020). Certainty of evidence was high. Similar results were reported in a network meta-analysis including 25,966 patients with T2DM, which compared GLP-1 RAs to placebo or no treatment (HR = 0.94, 95%CI = 0.76–1.16) (Zheng et al., 2018).

### Coronary Revascularization Procedures

Trials including LEADER, AMPLITUDE-O, and REWIND found no effect of GLP-1 RAs on the risk of coronary revascularization (Marso et al., 2016; Gerstein et al., 2019, 2021). In LEADER, 9,340 participants with T2DM and high cardiovascular risk were randomized to liraglutide or placebo (HR = 0.91, 95%CI = 0.80–1.04) (Marso et al., 2016). The AMPLITUDE-O trial of efpeglenatide (HR = 0.93, 95%CI = 0.69–1.26) investigated 4,076 participants with T2DM and a history of CVD or at least one cardiovascular risk factor in addition to kidney disease (Gerstein et al., 2021). The REWIND trial on dulaglutide (incidence = 6% for treatment group and 6.3% for placebo group; *p* = 0.46) included 9,901 individuals with T2DM (Gerstein et al., 2019). Meta-analyses on coronary revascularization are lacking.

### Chronic Coronary Syndromes, Vasospastic and Microvascular Angina

Existing evidence on the role of GLP-1 RAs on CCS and vasospastic and microvascular angina is scarce. The LIONESS study, a trial conducted among twenty-two patients with “chronic stable angina” examined the effects of subcutaneous liraglutide treatment on exercise-induced ischemia (Myat et al., 2015, 2021). After 3 weeks, liraglutide did not improve the change in magnitude of ST-segment depression at peak exercise during a sequential exercise tolerance test. Another study was conducted among thirty-three women without diabetes and with coronary microvascular dysfunction (Suhrs et al., 2019). Symptoms of MVA were measured by five dimensions—only the physical limitation score improved after 12 weeks of liraglutide treatment when compared to the control period.

## Conclusion and Future Perspectives

Coronary atherosclerosis, vasospasm of epicardial coronary arteries, and structural/functional changes in coronary microcirculation reduce coronary blood flow, which further results in ACS/CCS, vasospastic angina, and MVA, respectively. Despite existing controversies and the need for mechanistic insights from larger investigations, a number of studies suggest that GLP-1 RAs can exert antiatherosclerotic and vasodilatory effects in epicardial coronary arteries and improve CMF. Furthermore, GLP-1 RAs have beneficial effects for reducing the risk of MI or endpoints composed of coronary events (e.g., MACE, cardiovascular mortality), while solid evidence on CCS, vasospastic angina, and MVA is lacking. The observed beneficial effects of GLP-1 RAs likely translate into real-world settings, given that discontinuation rates may be lower for GLP-1 RAs than for other antidiabetic medications (Rea et al., 2021). Mechanistic evidence has been mainly derived from short to medium term studies, evaluating the direct effects of GLP-1 RAs on coronary arteries immediately after administration or after a few weeks of treatment, whereas evidence on clinical events is mainly obtained from studies with up to several years of follow-up. It is unclear if the mechanisms displaying instantaneous beneficial effects are the same ones that mediate the risk reduction in coronary events. Otherwise, alternative mechanisms such as glycemic control, weight loss, and reduction of blood pressure may be the main contributors in reducing the risk of coronary events. Furthermore, the efficacy of GLP-1 RAs has been mostly investigated among populations with T2DM and at high cardiovascular risk.

Additional investigations should focus on populations with optimal cardiovascular health in order to clarify the efficacy of GLP-1 RAs in primary prevention. Trials aiming for glycemic equipoise should appraise the long-term effects of GLP-1 RAs on coronary events independent of glycemic control. Possible effect modification by age, sex, diabetes status, glycemic control, obesity, CVD, and other comorbidities, as well as compounds, administration methods, and dosages of GLP-1 RAs should be evaluated. Finally, the combination of GLP-1 RAs with anti-inflammatory or antithrombotic medications can be a promising field to improve prevention and management of coronary events.
